# Refining Value Sensitive Design: A (Capability-Based) Procedural Ethics Approach to Technological Design for Well-Being

**DOI:** 10.1007/s11948-020-00223-3

**Published:** 2020-05-18

**Authors:** Alessandra Cenci, Dylan Cawthorne

**Affiliations:** 1 Department of Philosophy, Institute for the Study of Culture, University of Southern Denmark, Campusvej 55, 5230 Odense M, Denmark; 2 Unmanned Aerial Systems Center, Mærsk Mc-Kinney Møller Institute, Technical Faculty, University of Southern Denmark, Campusvej 55, 5230 Odense M, Denmark

**Keywords:** Value sensitive design (VSD), Procedural ethics, Amartya sen’s capability approach, Technological design for well-being, Democracy, Participatory-deliberative methods

## Abstract

Fundamental questions in value sensitive design include whether and how high-tech products/artefacts could embody values and ethical ideals, and how plural and incommensurable values of ethical and social importance could be chosen rationally and objectively at a collective level. By using a humanitarian cargo drone study as a starting point, this paper tackles the challenges that VSD’s lack of commitment to a specific ethical theory generates in practical applications. Besides, it highlights how mainstream ethical approaches usually related to VSD are incapable of solving main ethical dilemmas raised by *technological design for well-being* in democratic settings. Accordingly, it is argued that VSD’s ethical-democratic import would substantially be enhanced by the espousal of a *procedural ethics* stance and the *deliberative* approach to value and welfare entailed by Amartya Sen’s capability approach. Differently from rival ethical–political theories, its normative and meta-ethical foundations better handle human diversity, value-goal pluralism, conflicting vested interests as well as the epistemic-moral disagreements typical of contemporary complex democracies. Particularly, Sen’s capability approach procedural-deliberative tenets result in an “objective-impartial” choice procedure selecting a “hierarchy” of plural incommensurable values and rational goals thus, suitable to validate an applied science such as welfare-oriented technological design in concrete social environments. Conclusions suggest that refining VSD with a capability-based procedural approach to ethics fosters the concern for democracy and social justice while preserving vital scientific-technical standards. Major advantages are at an applied level to delivering ethically and socially justified, but yet highly functional technologies and high-tech products/artefacts.

## Introduction

Value sensitive design (VSD) is often regarded as one of the most promising ethical approaches in engineering and technological innovation.

Traditional design methods are known to have limited ability in creating ethical technology, since ethical aspects and social impact are not systematically or thoroughly considered during the design process. On the contrary, VSD is an interdisciplinary approach to the design of technology “that accounts for human values in a principled and comprehensive manner throughout the design process” (Friedman et al. 2013, p. 55). Its main mission is to supplement other design methods by including ethical values and social considerations as design inputs. In this sense, VSD represents a unique opportunity “to bring to the fore a proactive integration of ethics in the design of technology” (Van den Hoven 2008 In Jacobs and Huldtgren 2018, p. 1). This is achieved by setting up an integrative and iterative tripartite methodology, juggling and keeping in play the results of conceptual, empirical and technological investigations (Van de Poel and Royakkers 2011). Values and normative ideals stated in the conceptual and sometimes, empirical phases are supposed to have an influence on the technological phase—although, as evidenced by Van de Poel (2014, p. 20), the iterative process “is hardly deductive” and requires judgements and creativity on the part of the designer since “more than one specification is possible”.

In this paper, the possibility that values of ethical and social importance could meaningfully be translated into tangible, visible technological objects in consecutive iterations applying VSD is a prerequisite for technologies and high-tech products/artefacts to be considered *ethically relevant* or *socially justified*. It is taken as a point of departure to further address another crucial foundational issue underlying VSD: how to increase VSD ethical and democratic import by adopting a specific theoretical approach in contemporary ethical theory. These matters are elucidated at different but interconnected steps by answering two main questions:

Firstly, can technology and/or hi-tech products/artefacts actually embody values and ethical ideals, and, if so, how?

Main insights from the above analysis are further discussed to then provide a plausible answer to one of the most fundamental questions in ethical theory and, analogously, in VSD:

Secondly, how can plural, incommensurable values of ethical and social importance be chosen *rationally* and *objectively* at a collective-group level and then adopted either to substantiate technological design in democratic settings or to justify the implementation of novel high-tech products/artefacts in specific socio-economic-policy circumstances?

According to Ibo Van de Poel (2010, p. 6), proving that technologies and high-tech products could actually embody human values is one of the four main challenges of VSD. This issue is foundational to VSD but is rarely addressed empirically. This study aims to fill this gap by using the case of a civilian cargo drone to illustrate how ethical and social values can actually be *embodied technologically* in successive iterations (section "The Case: Insights from the VSD of a Humanitarian Cargo Drone"). This step of the argument (i.e., the case study) is important because, as shown by Winkler and Spiekermann (2018, p. 3), few VSD studies have reported iterations coming from promised enhanced designs incorporating values. It restates that technologies are not morally neutral but rather, a reflection and physical manifestation of specific values and normative goals since: “technical artefacts are not morally neutral because their functions and use plans pertain to the objectives of human actions, and those actions are always morally relevant” (Vermaas 2011, p. 19). These assumptions link VSD to mainstream *value-laden* approaches in the philosophy of science that, since the 90s, have attempted to establish that non-epistemic values (i.e., social-ethical–political-cultural values) have a pivotal role also in the inner stages of scientific inquiry and knowledge production, namely to engender good science and knowledge suitable for democratic settings (Longino 1990; Kitcher 2001, 2011; Douglas 2009, etc.). These approaches point towards overcoming not only the old value-free ideal (VFI) coming from neopositivism, but also more recent interpretations of scientific inquiry in which only epistemic values (i.e., epistemic norms, scientific standards) *directly* and *internally* contribute to the progress of science and to the achievement of scientific knowledge (Dorato In Machamer and Wolters 2004, p. 59). According to these views, non-epistemic values threaten the *integrity* and *epistemic status* of science and knowledge since highly detrimental to the *objectivity* required in scientific research and knowledge production; thus, they ought to be mere *external* constrains, mostly in science-related activities such expertise or public policymaking.

Recently, the importance of non-epistemic values also in the *internal* stages of scientific activity is becoming manifest in several research fields and disciplines, including in *applied sciences* such as engineering. According to Diekmann and Peterson (2013), non-epistemic values are essential components of engineering modelling. Nonetheless, authors warn that VSD, to be justified as leading value-laden approach in the field, needs to incorporate explicit considerations regarding the role of non-epistemic values in contributing not only to ethical-social objectives but also to pivotal epistemic-scientific goals. The practicability or relevance of including (non-epistemic) ethical-social-cultural values in *applied research* remains somewhat controversial,^1^ also due to a lack of empirical cases evidencing insights. In this optic, the contribution of the drone case (section "The Case: Insights from the VSD of a Humanitarian Cargo Drone") is twofold: a) it is an important testimony of the plausibility of the direct involvement of ethical and socially important values in the internal phases of scientific inquiry and reasoning (i.e., technological design) and scientific knowledge production in engineering (i.e., value-sensitive technologies and products/artefacts). (b) It illustrates that non-epistemic values can gainfully contribute to both epistemic and practical goals (i.e., delivering technologically valid-efficient products able to solve important social problems). Thus, by attesting that (non-epistemic) ethical-social values can actually be *embodied technologically*, the case study clarifies fundamental dilemmas of the general debate on values, science and democracy that are central to advance current VSD studies/practices as well. Thus, it can legitimately be considered a first original contribution of this paper to the existing literature in both fields. In particular, this close interconnection between normative and epistemic-technical goals is a prominent subject in nowadays philosophy of science but how these ideas reflect on VSD is an aspect largely overlooked. Even if rejecting the VFI, a radical *anti-positivism* attitude^2^ is considered inappropriate for an *applied science* field such as technological design, thus for ethical accounts of technology applying VSD, since other interpretations can be conceivable and more adequate. Here, a *post-positivist*^3^stance is accepted: while defending the importance of an ethical foundation (non-epistemic values), equal attention is devoted to the respect of vital scientific standards (epistemic values). In primis, *non-arbitrariness* in the selection of an ethical framework and *objectivity-impartiality* in the identification of values and normative ideals validating *bias free* value-sensitive technological design and human value-based technologies. Therefore, a primary task in this paper is to clarify whether and how (VSD-based) value-laden science and knowledge can be morally sound but also scientifically founded; namely, satisfactorily objective, cogent, reliable, able to circumvent bias and boosting evidence. It is argued that both (value) aspects can be fostered once the standard “morally neutral” VSD is complemented by espousing a *procedural ethics* stance (section "VSD and Ethical Proceduralism: Theoretical Insights and Practical Advantages") and a specific ethical framework consistent to it (section "Technology and Design for well-Being: Constructing “Value” by Adopting Amartya Sen’s Capability Approach").

These ideas conduct to another fundamental assumption in VSD: values need to be explicitly considered when designing or implementing technology since technological innovation may limit our power to perform certain actions or increase people’s power to perform others, thereby ensuring that “technologies can destroy certain values […] and make others virtually certain to be realized” (Tiles and Oberdiek 2005, p. 55). That is, technologies can produce and reproduce values that deeply affect people’s agency and positive freedom. According to leading VSD scholars, most misuses can be avoided by including “good” values as design inputs (Van de Poel 2014). It seems implicitly assumed that the *tout court* incorporation of values into the design leads necessarily to an ethically relevant technological embodiment that prevents, or at least makes it more difficult, to misuse a technology or a high-tech product/artefact. However, the fact that value-embodied technologies can be used in ways not intended by the designer is known, since “an artefact can always be put to different use to that which was originally intended, be misused or simply not used at all” (Vermaas 2011, p. 16). Designers and engineers may have, in fact, limited power of decision regarding implementation and final uses of their designed-constructed technologies and high-tech products/artefacts. These decisions most commonly rely on other stakeholders, such as funders or public authorities in the case of publicly funded projects. In a provocative article, Winner (1980) discloses that artefacts are never politically neutral. They have political qualities and it is possible to indicate ways in which specific features in the design or arrangement of a device or a system are used for re-establishing control and authority (p. 134). Therefore, good intentions on the part of designers or engineers—the ones applying VSD—could still be insufficient to produce a technological embodiment that is truly ethical and as such, avoid misuses or replication of patterns of power and domination in concrete settings.

What still remains rather unclear, also once applying VSD, is whether and how these “good” values can meaningfully be selected or *by whom*. Notoriously, people’s diversity, values and goal pluralism are main features of contemporary complex democracies and challenging for taking rational decisions at a collective level, since a variety of rational goals and vested interests of different stakeholders are inescapably involved. These issues are central in decision and ethical–political theory, and largely reflect on VSD, but the tripartite method offers no strategy to cope with main value dilemmas and plausible value disagreements. These shortcomings have been directly related to VSD’s lack of commitment to an explicit ethical theory (Jacobs and Huldtgren 2018). Indeed, the absence of clear and unambiguous ethical-normative principles or evaluative criteria determines a structural incapacity to discriminate between “good” and “bad” values, as well as to trade-off or prioritise among specific values and goals in case of conflict. Several practical problems have been related to VSD’s lack of an explicit ethical stating point and a missing precise methodology for identifying stakeholders (Manders-Huits 2011). The quantitative study recently made by Winkler and Spiekermann stresses (2018, p. 3) that in many VSD-based accounts, stakeholders are not accurately identified. Thus, a serious question arises: if stakeholders are poorly identified, who participates in the design process and whose values are embodied by existing technologies developed by implementing VSD? The data, in fact, suggests that, despite methods such as *participatory design, inclusive design* are frequently and intrinsically related to VSD, in practice, they are barely used in current technological design practices and even once applied, many relevant stakeholders are excluded (thus, their values remain largely unnoticed). What follows is that values of ethical and social importance behind many current VSD studies are either chosen *paternalistically*^4^ by designers, funders or they are designated by applying *pre-constructed* ethical-conceptual frameworks supposedly based on universal values.

A similar scenario draws attention to another eternal dispute in ethical theory that strongly comes across also in VSD: how plausible is it for ethical values to be universally applicable? Or, similarly, could values unrelated to contextual, social or historical circumstances^5^ be able to produce socially and ethically relevant effects in the real social world? What seems doubtful is that a VSD based on universal values or solely considering the values and goals of most powerful stakeholders could be adequate to generate truly “ethical” technological embodiments—namely *ethically relevant* and *socially justified* technologies and high-tech products/artefacts. In particular, the implementation of novel or contested technologies in concrete political-social-cultural-policy settings would demand greater involvement of the highest possible number of interested stakeholders and potential users-recipients (their plural values and goals), as well as fulfilling vital democratic desiderata.

Some main problems in practical applications (clearly illustrated by the humanitarian cargo drone case, this paper section "The Case: Insights from the VSD of a Humanitarian Cargo Drone") seem a product of fundamental *misconceptions* regarding the nature, scope of value-centred science and value-based technical design in democratic settings. Namely, its intrinsic *participatory-deliberative* character is evidently inconsistent with “front-loading" substantive ethics approaches based on fixed universal values that are most commonly used to substantiate VSD. As a solution, and the most original contribution of this study to existing literature, a moral foundation of VSD in a *procedural ethics* stance is provided by adopting Nobel prize-winning economist Amartya Sen’s *deliberative* version of the Capability Approach (section "Technology and Design for well-Being: Constructing “Value” by Adopting Amartya Sen’s Capability Approach"). Indeed, it is presumed that the “good” values suitable to substantiate technological innovation and value-centred technological design in democratic settings could hardly be chosen paternalistically since ethical relevance and ethical choices inevitably imply agents’ autonomy, positive freedom, freedom of choice and self-determination. Against the adoption of popular pre-constructed ethical frameworks based on universal values, the so-called objective list theories, a procedural-deliberative approach to “value construction” is alternatively adopted. Here, Sen’s capability approach normative and meta-ethical foundations are largely in keeping with an explicit and extended use of *participatory-deliberative* methods in the task of eliciting “shared” plural incommensurable values of ethical and social importance. Indeed, public debate and interpersonal rational scrutiny and deliberation involving the major possible number of “interested” stakeholders in the decision making is considered pivotal for ethical/normative choices and democracy. Thus, in selecting values and normative ideals underlying engineering and technological design practices, its adoption would favour the construction of a *top-down hierarchy of values*^6^ that could be more *agents-context sensitive* but still *satisfactorily objective*. Due to an equal attention to normative and practical issues, a similar approach is able to offer a *clear-cut* methodology for engineers/designers in concrete social and scientific contexts. Overall, this standpoint challenges mainstream ethical approaches validating or refuting technologies according to “a posteriori” ethical assessments of their uses or misuses, often carried out by applying pre-defined ethical frameworks. Conversely, it hints at a more dynamic, reactive and constructive role for ethics in which deliberatively chosen values and principled reflection are not mere add-on or “showstopper” to technological progress but its constitutive elements.

This paper develops as follows. By focusing on a re-interpretation in VSD terms of a recently operated humanitarian cargo drone (sections "Retrospective Analysis", "Prospective-Prescriptive Analysis"), shortcomings that can be related to VSD’s lack of ethical commitment are elucidated together with the ethical-democratic dilemmas connected to moral foundations of VSD espousing mainstream ethical approaches in contemporary ethics, particularly, objective-list theories based on universal values. In section "VSD and Ethical Proceduralism: Theoretical Insights and Practical Advantages", main insights of the former analysis are further discussed in relation to the challenges of either selecting/prioritising values and normative ideals for technological design in contemporary complex democracies. Hence, *ethical proceduralism* is indicated as the most fruitful ethical approach in similar circumstances. Then, in section "Technology and Design for well-Being: Constructing “Value” by Adopting Amartya Sen’s Capability Approach", the advantages for the so-called *technological design for WB* of supplementing VSD with Amartya Sen’s procedural-deliberative version of the capability approach are illustrated. This theoretical discussion highlights main normative and meta-ethical differences with other virtue-ethics approaches, including Martha Nussbaum’s theory of capabilities (2000), and stresses the insightful implications coming from Sen’s account preferential adoption. Finally, section "Conclusions and Future Research" re-states the overall advantages of a capability-based procedural VSD and indicates avenues for future research.

## The Case: Insights from the VSD of a Humanitarian Cargo Drone

In the following subsections, by applying VSD methods,^7^ the humanitarian cargo drone for transporting medical supplies and blood samples recently operated by the humanitarian organisation WeRobotics in Peru’s Amazon, between the city of Pucallpa and the remote village of Masisea (2018), is explored and then, redesigned.^8^

The cargo drone case was considered relevant for several reasons. It is a paradigmatic example of “applied” VSD while responding to the need of “civilizing drones by design” (Wynsberghe and Nagenborg In Di Nucci and Santoni de Sio 2016, p. 148). Even though VSD is not explicitly adopted by the drone creators, a set of ethical-social values undoubtedly motivate the original technological embodiment of the cargo drone prototype. In addition, the drone was operated in a challenging context and under extreme socio-environmental circumstances; thus, the case is expected to better highlight critical areas where an additional moral foundation (for VSD) would enhance practical applications. Precisely, subsection "Retrospective Analysis" explores which values and development goals guided or could have guided the first embodiment/iteration of the drone prototype. Then, subsection "Prospective-Prescriptive Analysis" illustrates how previously identified values and goals could better be embodied technologically by a subsequent iteration explicitly applying VSD: the drone second embodiment/iteration. Hence, this section initially elucidates how applying VSD retrospectively is suitable to uncovering the normative foundations of the original project. Yet, when VSD is applied prospectively-prescriptively, the emphasis is on its ability to deliver a new technological embodiment that might better align with previously identified values.

It is important to point out that the two analyses above and the case study more generally, are used with the only purpose of providing an *empirical starting point* for the theoretical-methodological discussion carried out in (sections "VSD and Ethical Proceduralism: Theoretical Insights and Practical Advantages"–"Technology and Design for well-Being: Constructing “Value” by Adopting Amartya Sen’s Capability Approach"–"Conclusions and Future Research"). The main investigative task is uncovering the shortcomings of an ethically uncommitted VSD in practical applications and suggesting how they could be solved. Thus, the case offers an insightful illustration of the argument supporting the suggested theoretical and methodological solutions. Consequently, the interest is not really about the empirical findings^9^ of the two analyses (although important)—i.e., supporting a set of specific values and goals for the civilian drone’s technology field. Rather, the attention is on how methodological-operational choices are made under VSD assumptions and elucidate their underlying rationale. Ultimately, a specific approach in contemporary ethical theory is indicated as the best way of supplementing/refining VSD in view of better coping with the epistemic-scientific and ethical-democratic dilemmas (evidenced by findings) commonly arising in applied sciences such as engineering and technological design in contemporary complex societies/democracies.

### Retrospective Analysis

A retrospective analysis is normally used to uncover and make explicit the implicit normative foundations of a project by focusing on the technological product itself. It belongs to the *conceptual* phase of VSD since is aimed at identifying designers and/or other stakeholders’ inputs and consider how they impacted on the technological phase of the hi-tech product in question.

The humanitarian cargo drone retrospective analysis relies on existing literature on the case; primarily, a technically focused report (Meier et al. 2018). Then, since no empirical strategy to elicit values is described, for instance, by involving the local population; the company’s mission statement and its self-declared development goals are used to extrapolate the foundational values of the original project (WeRobotics 2019).^10^ According to the company’s website, WeRobotics aims at “using robots and drones to improve the lives of people in emerging economies”. Its (professed) main aim is to develop “robotics for the benefit of all”. This endeavour is fundamentally attached to the development of “sustainably localiz[ing] appropriate robotics solutions that in low-income countries serve to accelerate the positive impact of aid, health, development and environmental efforts”. Also, it is overtly affirmed that WeRobotics’ actions “strive to contribute to United Nations Sustainable Development Goals” (UN SDGs 2019). Therefore, a set of explicit values and goals supposedly enhancing *human development* and *people’s welfare*, can be identified:

- "benefit of all" = general interest, fairness-equity, universal usability:
- "sustainably" = economic efficiency, environmental sustainability;
- "aid" = cooperation, solidarity, humanitarianism;
- "development" = Physical-mental and material welfare;
- "environmental effort" = environmental sustainability;

The emphasis on UN SDGs is recurrently pointed out, also by saying that the development of disadvantaged (low-income) local communities (localize) is closely related to technological progress, and that the improvement of human welfare is closely tied to the possibility for local communities to be more “autonomous” and to develop an own “identity” with the help of the technological innovation. Plainly, a set of main goals emerge:

- Goal 3: good health and well-being for people = physical and psychological welfare;
- Goal 9: industry, innovation, and infrastructure = material-economic welfare;
- Goal 10: reducing inequalities = fairness-equity;
- Goal 11: sustainable cities and communities = environmental sustainability;
- Goal 13: climate action = environmental sustainability;
- Goal 15: life on land = environmental sustainability;

This simple VSD-inspired conceptual analysis reveals the values and goals that most likely, has been *embodied technologically* by the company in the prototype cargo drone (first iteration). As they have been derived from universally valuable UN SDGs, the procedure can be considered as equivalent to the application of a substantive ethical theory based on an “objective list” of universal values. Nevertheless, the crucial issue here is not which specific values arose from the retrospective analysis, but *to what extent* considerations about values and normative goals influenced the technological phase (a main assumption of VSD). Although VSD does not provide a systematic way to rank different values, the drone’s primary function is transporting blood samples (fast and cheap) to the hospital in order to have earlier results and reduce diagnostic time. In this optic, enhancing the health outcomes (i.e., physical-psychological welfare) of the local population could be considered a primary goal and the human value with the highest importance while economic efficiency and environmental sustainability come immediately later. Thus, by supposing a direct impact of the conceptual upon the technological phase, physical-psychological welfare, material welfare and economic efficiency as well as environmental sustainability (in this order) should be considered the main values behind the technical solutions adopted by WeRobotics.

First, enhancing the ideal of *physical-psychological welfare* implies increasing the healthcare service ability while preserving safety. In these tasks, the drone type chosen by WeRobotics is acknowledged for increasing safety and reducing risk. Moreover, the implementation of the drone is expected to reduce diagnostic time—by allowing faster transportation and earlier examination of blood samples—from between two and twenty-eight hours to a few hours in total. Thus, in all cases where there would be no need for further physical transportation of patients to the hospital, the use of the cargo drone has potential for increasing people’s opportunities for welfare. Yet, drones are not only faster, but also significantly less costly than the ordinary boat service. Hence, economic efficiency, another pivotal value and goal in WeRobotics’ list, could be supported once the current transportation is replaced with a drone delivery service.

Second, even though enhancing economic efficiency in this context means reducing costs compared to the boat service, several trade-offs appear once considering also the value of *material-economic welfare*. The WeRobotics drone is designed and manufactured in the USA but it has been slightly modified to align with the company’s main values and goals: by substituting the camera with a transportation device for the blood samples. Anyhow, the economic paybacks in loco are restricted to those for the drone operation and maintenance. Besides, local stakeholders such as charter boat operators would be evidently harmed financially by the implementation of a cargo drone service in the ordinary diagnostic activities. While imagining a (long-term) future in which drones could also be used to transport people, also public decisions regarding infrastructure investments in the area could be seriously affected. For instance, roads and bridges connecting smaller villages to larger cities could not be built anymore. Thus, properly addressing these socio-economic challenges from an ethical perspective, it demands additional knowledge of the local communities’ productive conditions, their vested interests since they should be reconciled.

Third, enhancing the value of *environmental sustainability* requires the preservation of nature and minimisation of pollution. A drone impacts the environment during its entire life cycle (e.g., extraction of building materials, manufacturing phase, operation-functioning phase, etc.). Yet, the environmental impact of the river boat service is significantly higher. The drone is—at least for final users—significantly more environmentally sustainable than the conventional transportation method. Thus, if environmental sustainability were the leading value, irrespective of any other plausible welfare consideration or socio-economic risk, a cargo drone service ought to be implemented. But also, these considerations seem strictly dependent on knowing more about all local stakeholders’ precise values, goals and vested interests—for instance, their willingness to sacrifice some immediate welfare benefits with the view of protecting the natural environment for future generations.

Therefore, once applying VSD methods explicitly, there are no doubts that WeRobotics’ values and goals mirror the main technological choices when developing and technologically embodying the cargo drone prototype. As expected, the setting in which the drone was operated and the extreme socio-economic consequences of implementing a cargo drone service are helpful to highlight that novel technologies could have a *huge impact* on ordinary people’s lives and welfare. Although values of ethical and social importance clearly influenced the building of the cargo drone, is also obvious that many local stakeholders’ values remain unnoticed. That locals had no voice in the design and building of the WeRobotics drone is confirmed by the circumstance (clearly said in the technical report) that the company used a standard model manufactured in the USA and adapted for the occasion.

This is problematic since only supporting the values and goals of the company or the most powerful stakeholders could be insufficient to enhance the well-being of substantial parts of the local population, or to advance vital ethical and democratic ideals such as people’s agency, freedom, autonomy, social justice as well as transparency, legitimacy and accountability of public decisions. The drone case similarly suggests that invoking universal values and goals (i.e., UN SDGs) cannot adequately cope with values and social dilemmas that might arise when implementing novel technologies in concrete political-socio-economic circumstances. Indeed, technological decisions can hardly be separated from other public deliberations and in this optic, local communities and local stakeholders’ values, goals, vested interests ought to be made explicit, balanced and then accommodated by a proper VSD.

### Prospective-Prescriptive Analysis

As evidenced in the former section, a VSD-inspired retrospective analysis is able to uncover hidden values of ethical and social importance in existing technologies and show that there is a close interaction between VSD’s conceptual and technological phases underlying the technological embodiment of high-tech products/artefacts. This relation is substantially strengthened in a prospective-prescriptive analysis since here, VSD’s main aim is to look for the best possible alignment between—previously identified—ethical values, normative ideals, goals and certain technological solutions.

Still, in order to develop a new drone—with a different technological embodiment—additional assumptions—external to VSD—regarding the relative importance of formerly identified values and goals are needed. A prospective-prescriptive analysis suitable to guide scientific-technological choices *necessarily demands* ranking values and goals by establishing clear priority-setting rules. Fundamentally, disentangling value conflicts or discriminating among incompatible goals in order to establish which value or goal must prevail when looking for a precise technological solution (i.e., a product with specific characteristics) demands adopting a precise normative position. It guarantees that, at the end of the process, a final technological embodiment—namely, a concrete high-tech product—can be created. Following Cawthorne and Cenci (2019), technological choices guiding the second possible embodiment/iteration of the drone are defined by applying a *maximising approach* (but other possibilities are also plausible). Thus, WeRobotics’ values and goals are ranked as follows: physical welfare is assigned the highest priority, while material welfare-economic efficiency and environmental sustainability are, respectively, in second and third place. In the following description, technological choices (and their rationale) are illustrated in relation to the ability of specific technical solutions to realise previously identified values (of WeRobotics!) and produce a value-sensitive technological embodiment (that could be ethical or not).

First, maximising the value of *physical welfare* demands further improving the healthcare service ability of the drone: its rapidity and reliability but also safety in transporting the blood samples. Obtaining a new drone twice as fast as the WeRobotics’s prototype and equally reliable in delivering the samples seems possible but it would have a greater environmental impact (cf. pp. 1122–1125). This is a clear value conflict: the value of physical welfare and environmental sustainability are in opposition. Though, by following the previously established priority setting rule, the proposed technical solution (i.e., an internal combustion engine instead of electric motors) delivers a faster drone that, while much more sustainable than the river boat, is still less sustainable then the original prototype.

Second, maximising the value of material welfare in this context could refer to boosting economic efficiency and increasing the financial benefits for the highest possible number of local stakeholders. For example, promoting higher-quality employment in the area by increasing local design, manufacturing and maintenance (e.g., change the type of automation) could be functional to create employment opportunities in the area. Major both technical and social advantages would come from local designers increased knowledge of the environments, local communities and their needs. Likewise, rather than utilising an off-the-shelf automation software and automated support infrastructure, the use of alternative technologies involving an additional human control is known to increase safety and reliability of operations.

Third, maximising environmental sustainability of the drone service in this context could mean having a strong concern for the preservation of nature and/or minimising pollution. Even though the prototype drone is much more sustainable that the boat service, additional considerations about the end-of-life of the drone (Fiksel 2009) can be addressed by re-designing the drone with components that can easily be exchanged, disassembled and recycled.

Therefore, priority given to one value/goals or another might result in a technological embodiment for the cargo drone (second iteration) that differs in subtle but critical ways from the original prototype. The drones obtained by applying a “maximizing” VSD prospectively-prescriptively can be *twice as fast* as the original prototype and, due to the technical solutions adopted, also *safer* (physical welfare). Further assuming that the drone can be redesigned and manufactured locally, high-quality employment in the area would be created (material welfare)—for instance, by adding an extra human control device (also beneficial to increasing reliability and safety of operations) or, by using modular components that could easily be replaced, exchanged, recovered and then recycled when the drone reaches its end-of-life (environmental sustainability). Thus, once applying VSD methods prospectively, the foundational values and goals declared by WeRobotics company/team could better be realised and, plausibly, more desirable social effects could be achieved. Nevertheless, the prospective analysis substantially reveals that even the values and goals of a single stakeholder (WeRobotics) can be *inconsistent*. It is a problem since it seems evident that the technological embodiment of one value/goal or another lead to rather different technological products.

That the standard “morally neutral” VSD cannot deal with value pluralism, solve value conflicts or indicate how to trade-off is known. But what the drone case clearly highlights is that an *external normative criterion*, distinguished and distinguishable by the VSD procedure itself (i.e., the priority rule), is necessary to disentangle crucial value dilemmas and to get a definite technical outcome: a technological embodiment that meaningfully reflects leading values/goals. Remarkably, an even more critical (unsolved) ethical issue become evident: the values and goals of other stakeholders, perhaps substantially different from the ones of WeRobotics company/team, couldn’t be used to design and technologically embody both versions of the drone.

Even after applying VSD explicitly, the main ethical challenges for final users/direct stakeholders’ agency, positive freedom, self-determination as well as the threat to democracy namely, to legitimacy, transparency, accountability of selected values and normative ideals underlying the technological design of the two drones, largely persist. This is due to the normative views adopted to inform and substantiate both VSD analyses from an ethical standpoint. Retrospectively, a set of supposedly universal values and goals inspired by the UN SDGs has been assumed (as typical in liberal objective-list ethical theories). Prospectively, in order to identify precise technological solutions, a maximizing approach (as typical in utilitarian ethics), has been used to prioritize and rank previously identified values. Thus, it seems that the possibility of achieving better ethical-democratic and scientific-technical outcomes—namely, increased precision, accuracy of VSD-based technologies obtained but especially, an expansion of the participation to a larger number of interested stakeholders to the design process—depend not only on a moral foundation of VSD but substantially, on the adoption of a *specific normative position* that could be able to support both instances.

In order to cope with the critical issues above and providing a *superior moral foundation,* the following sections propose to supplement/refine VSD by adopting a procedural ethics stance and consistently, Sen’s deliberative version of the capability approach. This normative view is also external to VSD but is not *chosen arbitrarily*^11^ thus, any *relativist objection* is avoided (i.e., why this ethical view and not another?) and improved epistemic and social effects are expected. The stress is on the great potential of a similar combination to most profitably handle the challenges raised by human value-based, welfare-oriented technological design in democratic settings.

## VSD and Ethical Proceduralism: Theoretical Insights and Practical Advantages

The former section pointed out how in many VSD studies a fundamental (unaccomplished) “ethical task” is about identifying a normative view suitable to better inform—*unambiguously* and *non-arbitrarily*—technological design practices in concrete social settings. Before illustrating this paper’s proposal, discussing further some key critical issues in practical applications can be useful to better understand what is at stake and the scope of the proposed solutions (sections "Technology and Design for well-Being: Constructing “Value” by Adopting Amartya Sen’s Capability Approach", "Conclusions and Future Research").

As shown by the WeRobotics cargo drone case, even a small village can represent a *pluralistic* range of values. Even so, perhaps for simplicity, it is not infrequent that values and goals that matter and are considered when applying VSD, are selected *paternalistically* by designers or powerful stakeholders and then, further justified by evoking pre-defined ethical frameworks based on supposedly universal values. Another problem evidenced by the VSD retrospective-prescriptive analysis is that *subjectivity* or *arbitrariness* in prioritizing previously identified foundational values are impossible to evade without assuming a precise normative position*,* and similarly, that not every normative standpoint is adequate. An (unanswered) ethical question is: can (whatever) human values really be universal? For example, the UN SDGs are often considered to contain non-controversial values; though, as pointed out by MacIntyre (2013), the United Nations does not provide any reasons or specific philosophical ground to justify why these particular values and goals should be generally endorsed. The list of twelve human values enhancing engineering and technological design suggested by Cummings (2006)^12^ is another example of the same problem, which in ethics is common to many objective list theories.^13^ Most likely, no technology can satisfy everyone’s values and interests at the same time. But drones, like any other technological product strongly impacting people’s lives and welfare, should not be imposed without proper public discussion and deliberation aimed at achieving, eventually, local communities’ explicit consent. Conceivably, locals would rather have roads and bridges built, instead of a cargo drone delivery service. Obviously, whether the design or the implementation of a technology is enforced, it can hardly be considered truly ethical or socially justified (no matter if VSD is carried out or not!). Taking rational decisions at a collective level entails empowering local communities; thus, enhancing people’s agency, positive freedom, control, self-determination would inevitably suppose larger participation to the decision making*.* Accordingly, a VSD leading to a genuine *ethical technological embodiment* implies that the major possible number of interested stakeholders, their plural values and goals should be meaningfully elicited, balanced and then accommodated.

Noticeably, VSD’s empirical phase is highly *context specific*. In order to profitably cope with the possible negative social impact of a technology on a specific group of people or on a specific environment, in-depth knowledge of local communities, all stakeholders’ conflicting values, goals and vested interests is desirable (e.g., a stakeholder analysis,^14^ an environmental risk and social impact analysis and so on). Perhaps, a “perfectly ethical” technology does not even exist or could be prohibitively expensive to produce. Nonetheless, increasing this possibility entails that the trade-off amongst agent’s conflicting values, goals, vested interests as well as between material-human costs or efficiency-equity considerations should actually be performed thus, paternalistic solutions should be avoided. Major problems are in relation to *transparency*, *legitimacy* and *accountability* of values and goals chosen to substantiate VSD-based technological design. Often, drones are announced as able to eliminate “dull, dirty or dangerous jobs” (Finn and Wright 2012 In Di Nucci and Santoni de Sio 2016, p. 148). But there could be occasions in which implementing a drone delivery service can be socially unfeasible if it proved to be too controversial or too highly detrimental for one or more local stakeholder. It is not difficult to imagine a hard case in which 80% of the local population is financially related to the boat service (manufacturing, transportation, logistics, etc.); thus, implementing a drone delivery service could leave most of the locals without the essential means of living. Despite reducing costs and producing some evident benefit for human welfare, a drone delivery service could hardly be implemented in similar socio-economic circumstances. Therefore, as insightfully illustrated by the drone case, the main ethical task relies on finding how a larger variety of stakeholders’ values and goals could be better supported by applying VSD or, alternatively, why only some of them should be prioritised in case of conflict and in the view of superior collectively chosen objectives, the general interest. The leading moral dilemmas that arise in contemporary complex democracies can be re-proposed in the following terms: how/to what extent can value conflicts be solved not paternalistically and, the concern for value pluralism enhanced while applying "bottom-up" ethical approaches to the justification of values and normative ideals underlying applied sciences and technological design in concrete social environments?

It seems that obtaining *ethically relevant* and *socially justified* technologies strongly demands that VSD might incorporate an *objective-impartial*, *non-arbitrary* nor *paternalistic* ethical procedure to identify “shared” normative ideals and “public” values and goals adjusted to specific technological cases and social circumstances. Here, the plural values, goals and vested interests of a large variety of diverse stakeholders could be meaningfully identified, and in case of conflicts, properly opposed, traded off, negotiated, ranked (to further prioritise some of them). This is in keeping with Manders-Huits’s arguments (2011, p. 271) that a VSD suitable to deliver ethical high-tech products or perform proper normative evaluation of technology needs an explicit and justified ethical starting. Likewise, it substantiates Van Wynsberghe’s and Robbins’ (2014) claim that validating VSD as the leading conceptual framework for technological design requires a “robust” ethical-philosophical underpinning (now absent). It is believed that a normative foundation able to deliver a similar “robust” ethical procedure for VSD is attainable once espousing a procedural ethics stance and consistently, Amartya Sen’s procedural-deliberative version of the capability approach. This solution would better handle the ethical dilemmas attached to a morally neutral VSD while evading the flaws for democracy that rival ethical views, most typically attached to VSD, are intrinsically unable to solve. Perhaps more importantly, validating scientific and technological decisions in democratic settings entails coping with human diversity, value and goals pluralism but also with insoluble moral dissents, often leading to important scientific-technological disagreements and epistemic bias. In an applied science domain as technological design, the delivery of “truly” ethical technology and high-tech products/artefacts seems to rely substantially on the possibility that VSD can involve a *plurality* of (non-epistemic) values and goals for action without losing its scientific appeal. That is, an attention to ethical-social values and similarly, to essential *scientific standards* and *epistemic ideals* (objectivity, generalization potential, flexibility, versatility etc.) is pivotal. These concerns were essential for VSD creators, in fact, are reflected by the preference for an ethically uncommitted VSD. Similar apprehensions have been progressively lost in the attempt of providing an ethical foundation, mostly for practical purposes, typically by linking VSD to specific ethical values and normative principles. Though, most of existing VSD-based accounts can be criticised for an *unjustified* prime focus on ethical-social values and the scarce attention they pay to the scientific-epistemic implications of attainted value-oriented disciplines, value-based technologies. Namely, consequences for the required epistemic status of applied sciences (i.e., rational and objective technological design practices) thus, for the validity and reliability of the scientific knowledge obtained (i.e., bias free technologies and hi-tech products).

It is believed that important scientific and societal goals would simultaneously be achieved once adopting a *procedural*, rather than a substantive approach to ethics and value. The attention for the “correctness” of the choice procedures, instead of merely supporting explicit values and normative ideals, oblige to be *rigorous* in the setup of the deliberative scenario and the choice of applied methods (since the values underlying technological design should be selected *empirically*). In this view, (non-epistemic) ethical-social values, normative ideals and goals underlying VSD—to be transparent, legitimate and accountable—demand to be designated by means of public debate, interpersonal critical discussion and deliberation involving the highest possible number of interested stakeholders: designers/engineers, public institutions, private companies, local communities, public authorities etc. That is, valid and reliable human value-based applied science and technologies ought to be informed by a plurality of values and goals that could be *self-chosen* and *self-determined* by autonomous moral agents and elicited by means of participatory-deliberative methods. This procedural-deliberative approach to “value construction” avoids the scientific-technological and ethical paternalism involved by mainstream ethical theories and is able to circumvent important epistemic bias. Precisely, it does not imply embracing subjectivism or ethical relativism, as is often erroneously believed. This is conceivable by further assuming the possibility of *rational interpersonal agreements* regarding the reasons underlying a plurality of foundational values and rational goals of action, as well as the existence of a *public practical reason* able to profitably orient rational social choices and collective action (more details in the next section).

Many contemporary ethical–political theories give primary importance to human welfare but substantially disagree on how well-being is defined and measured. Mainstream ethical approaches often indicated as suitable normative grounding for VSD (i.e., liberal-egalitarian theories providing objective lists of universal values), offer substantive descriptions of the aspects of ethical importance relevant for well-being by exclusively relying on the *reasoning of theorists* and disregarding any cooperative-participatory-democratic ideals. For instance, a recently published VSD study suggests solving value conflicts behind the adoption of contested technologies by focusing on the development of “perfectly just” institutions (Dignum et al. 2016). It implicitly adopts the contractarian view known as institutional transcendentalism (Rawls 1971 In Sen 2009, Ch. 2), whose capacity to successfully manage value and goals pluralism, as well as epistemic-moral disagreement, is frequently questioned (see also Nussbaum 2006, Ch. 1). Besides, the situation described by the study—all stakeholders in the Dutch society seem to have the same values concerning the technology in question—could be rather difficult to reproduce in bigger nations or in less socio-culturally homogenous societies. Main critical aspects of a similar ethical posture concern the paternalistic method used to select values, normative ideals and principles of justice but also, the rigidity of the objective list of supposedly universal values obtained. All these difficulties would be solved when complementing/refining VSD by committing to ethical proceduralism, and consistently, to Amartya Sen’s deliberative version of the capability approach; namely, one of the most important *procedural theories of well-being* in the contemporary liberal egalitarian tradition. This standpoint is expected to be especially commendable in the so-called *technological design for well-being* (sections "Technology and Design for well-Being: Constructing “Value” by Adopting Amartya Sen’s Capability Approach"–"Conclusions and Future Research"). Indeed, Sen’s capability approach (1999) is known for more successfully dealing with value pluralism, conflicting vested interests while solving critical democratic shortcomings (Comin 2018, Cenci forthcoming). This version of the approach primary attention to value and goals pluralism is represented by the “multidimensional welfare” concept and the “ethics and economics” paradigm, both pioneered by Sen in Welfare Economics (Sen 1985, 1987). It represents a fruitful normative basis to substantiate VSD studies in democratic settings since offers a broader understanding of human well-being as *human flourishing* but further combined with a procedural-deliberative approach to its determination and measurement. Another well-known peculiarity of Sen’s approach (2000) is that is jointly grounded on *ethical-deontological* and *economic-consequential* evaluation. That is, by profitably reconciling vital normative and epistemic-pragmatic considerations, is able to answer both *efficiency-efficacy* and *fairness-equity* questions. Hence, objectively-impartially selected values and principles as well as their actual fulfilment are tested on the basis of the outcomes produced once implemented at a societal level. This establish a tangible difference with rival ethical approaches (e.g., utilitarianism or egalitarianism), which instead *unilaterally* emphasise one aspect or another (i.e., economic efficiency or equality). The theory of justice grounded on Sen’s capability-based conceptual framework, also defined as *capability justice* (Sen 2009), is often understood as a viable alternative to utilitarian theories and a broader view in the contemporary liberal-egalitarian thinking (see Kauffman 2005).

As a final remark, the solution for VSD defended in this paper cannot be confused with the capabilitarian accounts defended by Oosterlaken (2015) and others in technology and design for well-being, which most frequently rely on philosopher Martha Nussbaum’s deontological and complete list of ten valuable capabilities (2000, Ch. 2). Although, in both versions, there is wide agreement on the importance of technological progress to advance human capabilities and the relevance of welfare-oriented technology and design for human progress, it is maintained that Sen’s *deliberative-informational* version of the capability approach can better address neglected but crucial aspects involved in validating virtuous engineering and technological design practices in democratic settings. Fundamentally, its adoption provides a more positive answer to Pols and Spahn’s (2015) sceptical conclusions regarding whether design methods that seek to promote democracy and justice in the design process (participatory design, VSD, inclusive design etc.) can succeed in their mission. Once Sen’s procedural-deliberative capability approach is used to complement/refine VSD, many current problems of existing ethical design methods and practices are eluded since, contrarily to what is usually done, there is an explicit focus on *theories* rather than specific *values.* As inherent to a procedural ethical theory, the main investigative task—when searching for *design guidelines* suitable for engineering, technological progress sensitive to democracy and social justice ideals—is not to provide a definite, complete list of universal values and normative principles; but rather the emphasis is on the *transparency*, *correctness* and *inclusiveness* of the ethical procedure behind the choice of foundational values and goals underlying technological design for well-being in concrete socio-cultural-policy environments.^15^

## Technology and Design for well-Being: Constructing “Value” by Adopting Amartya Sen’s Capability Approach

Although several economic and ethical–political theories that have been reasonably defended assume that enhancing human welfare is a central goal at both the individual level and the societal level, there is substantial disagreement regarding how well-being is defined and measured. Different theoretical approaches prioritise different aspects of well-being and rely on rather different normative and meta-ethical assumptions to establish the importance that specific environmental-social-policy aspects, values and goals have or should have for people’s welfare achievements or their opportunities for welfare.^16^ This section concentrates on vital foundational aspects related to *value construction* in democratic settings and elucidates how the adoption of Sen’s approach, which is also conducive to the use of specific empirical methods, is best suited to grasp contextualised but objective plural values of ethical and social importance for well-being research in the technology and design field.

The insights of the so-called *design for well-being*, particularly the ones coming from the adoption of a virtue-ethics approach and, explicitly, of the capability approach as a broader normative view in the field suitable to advancing human welfare throughout capabilities-oriented technology and design, has been often evidenced in the existing literature (Steen 2016; Oosterlaken 2015). Thus, further endorsing a capabilitarian perspective against rival theoretical views (e.g., utilitarian theories, liberal theories of justice) or elucidating its main concepts (i.e., capabilities, functionings, conversion factors) is not what mainly motivates the theoretical discussion carried out in this section. The focus is how opposing assumptions underlying the different interpretations of the capability approach (Nussbaum’s and Sen’s) impact operationalisation strategies and the choice of experimental methods applied either to eliciting foundational values (i.e., a set of valuable capabilities) or to establishing rational goals to be pursued at a societal level (i.e., social/public value). Similar issues are still slightly under-researched but fundamental to validating the capability approach as suitable philosophical ground for either VSD or other methodologies used in applied sciences such as engineering and technological design.

A significant exception to the above is represented by Steen (2013, 2016), who explicitly associates methods such as *participatory design* with the capability approach’s tenets and stresses how crucial ethical virtues for human development—namely cooperation, curiosity, creativity, empowerment and reflectivity—can be enhanced by technological design procedures based on its espousal. However, some more substantial clarifications are needed since the two versions of the approach are substantially different from a normative and meta-ethical standpoint.^17^ What remains unclear is how Nussbaum’s deontological, expert-led, over-specified, complete and perfectionist list of ten basic capabilities (not openly rejected by Steen) could be in keeping with the participatory-deliberative ideal and inherently contextual character of similar investigative methods.^18^ Differently from Sen’s open normative framework, Nussbaum considers capabilities, and suggests using them, in the same way as Rawlsian primary goods—namely as a “currency” of basic universal entitlements in which little space is left for public discussion, rational critical scrutiny and deliberation (Nussbaum In Kaufman 2005, Ch. 2). Properly discriminating among the theoretical foundations of the two approaches is pivotal in practical applications since the adoption of one capabilitarian version or another implies the use of dissimilar (ethically sensitive) experimental techniques that point to very different kinds of (value-laden) experimental knowledge. In this vein, the normative and meta-ethical foundations behind the two versions of the capability approach are explored (in occasion, against rival theories) and the advantages of providing a moral foundation for VSD in a procedural-deliberative stance by adopting Sen’s interpretation is evidenced. The emphasis is on Sen’s capability approach’s ability to cope with the so-called *moral overload*^19^described by Van den Hoven and colleagues (2012)—precisely, how main moral dilemmas could most profitably be re-addressed by a refined VSD favouring a normative structure based on a *procedural* rather than the substantive approach to ethics and value typically espoused in ethical–political theory, including Nussbaum’s capabilities approach. Differently from mainstream ethical–political theories in which valuable ethical objects and goals are established (paternalistically) by theorists, Sen’s capability approach procedural-deliberative tenets entail setting up functional empirical strategies to identify and appropriately aggregate/trade off conflicting values, goals and vested interests of the different stakeholders. For this reason, it is believed to indicate precise practical-operational solutions to evade the empirical challenges—*epistemological* and *aggregation* challenges—rightly evidenced by Van de Poel (In Oosterlaken 2015, pp. 233–238) as main unresolved flaws of current capability-based attempts of operationalisation. An important empirical challenge is about setting up operational strategies suitable to reconciling both scientific-technological and ethical-social desiderata. In brief, coping with the foremost experimental challenges related to the process of “value construction” in concrete settings, including evading the so-called *naturalistic fallacy*, demands an increased consideration for stakeholders’ participation and empirically derived values. Hence, applied methods should be able to deliver normative principles and ideals representative of the reconciled, mediated values and goals of experts-engineers/designer, public authorities, the local communities affected by technological innovation. Specifically, applied studies informed by *participatory-deliberative* methods and by the latest capability-based methodological advancements in the field of welfare analysis and economic evaluation can be highly illustrative of the normative, epistemic but also, practical insights of Sen’s procedural-deliberative approach, instead of *tout court* applications of Nussbaum’s list (I will return on this later).

What is important to first point up is that the capability approach (in both versions) is a *middle-level theory* based on middle-level ethical principles (i.e., capabilities); thus, it stands between unconditional principles and context-specific obligations. Following Diekmann (2013) and, Jacobs and Huldtgren (2018), a commitment to a similar ethical standpoint is desirable to cope with the practical problems that VSD encounters when aiming to provide more systematic approaches to ethical engineering and technological design. What Sen’s approach insightfully adds to the discussion is that the mid-level principles (i.e., valuable capabilities or, similarly, social/public value), to be considered really adequate from an ethical standpoint, ought to be chosen, prioritised and traded-off *intersubjectively* through open public debate, critical discussion and deliberation (Sen 2004). The result is that ethical principles guiding VSD practitioners, to be valid from an ethical-social point of view, should be corroborated by a (rational) *deliberative ethical procedure* selecting values at a collective-group level. It would enhance not only individual agents’ freedom of choice, autonomy and self-determination, but also empathy, reciprocity and cooperation. This can ultimately guarantee that selected ethical objects—the valuable capabilities—could be a genuine expression of the plurality and incommensurability of different stakeholders’ values, preferences and rational goals for action in specific social settings. These assumptions underlying Sen’s capability approach openly challenge standard welfare economics’ ethical and methodological individualism,^20^ in which value and even social value, is obtained by means of eliciting methods (highly vulnerable to the naturalistic fallacy) targeting individuals’ preferences then, merely sum-aggregated to get a collective/social standpoint. Similarly, they entail a rejection of ethical frameworks based on a set of untested universal values,^21^ which is typical of objective list theories in which value and social value are identified mostly by means of logical-conceptual analysis by philosophers and ethicists or, ideologically, by politicians or policymakers.

In particular, the different way in which Sen’s procedural-deliberative and informational version of the capability approach is able to cope with three main objections to objective list theories of well-being pervading the contractarian tradition (Rice 2013) can further elucidate why it represents a real alternative and most plausible solution to evading the shortcomings of subjectivist utility-based accounts and methods extensively used in economic evaluations of well-being (the dominant approach in the field). The main challenges/objections are:

- How *pluralism* about well-being can better be articulated;
- How *objectivism* about well-being can better be achieved (i.e., context-agent sensitive but satisfactorily objective value judgements);
- Whether/how *plural*, *incommensurable* values and goals can be empirically selected by satisfactorily reconciling oppositions and disagreements.

These three points are addressed by further highlighting methodological and empirical-operational implications.

First. Once adopted to substantiate VSD, a procedural value theory enhances not only pluralism about well-being, but also agency, autonomy and the self-determination of stakeholders since technological development and innovation could be based on a plurality of self-chosen values and rational goals of action that target concrete problems and specific needs. As often pointed out by Sen (2004, p. 78), the emphasis of mainstream objective list theories such as Nussbaum’s or Rawls’ universal, over-specified and complete lists of valuable objects for well-being (even capabilities) are insufficient to solve specific problems in concrete political-social-cultural-policy settings. Multidimensional and incommensurable qualitatively different aspects of well-being that are elicited by means of theorists’ normative reflections and detached from contextual and social circumstances, since the recipients of the policies or political actions are excluded by the decision-making, can hardly generate truly ethical decisions. Equally, endorsing a set of (supposedly) universally good values, as most leading deontological and liberal-egalitarian ethical–political theories do, is not satisfactory since values or goals can change substantially in different settings and over time. As the drone case study shows (this paper, sections "Retrospective Analysis" and "Prospective-Prescriptive Analysis"), although multiple (non-epistemic) values can be embodied technologically, it cannot resolve a preliminary matter: technologically embodied values, to be really ethical, ought to also reflect the point of view of local communities and not merely of theorists, designers or the most powerful stakeholders (e.g., the drone’s creators). In this scenario, Nussbaum’s theory of capabilities encounters the same problems of any other objective list theory (e.g., UN SDGs). Indeed, the main ethical task does not rely on identifying a list of universal ethical values and goals but on avoiding ethical and scientific paternalism by guaranteeing the *correctness* of the social choice procedure behind the selection of plural and incommensurable values of ethical and social importance—precisely, by extending *participation* and *engagement* to the decision-making. This is precisely, Sen’s open framework most positive contribution: its procedural-deliberative tenets are crucial to achieve ethical-democratic goals such as enhancing stakeholders’ agency, positive freedom, self-determination as well as to boost transparency, legitimacy, accountability of both the ethical procedure and the chosen values and normative ideals.

Second. The procedural-deliberative tenets underlying Sen’s account are structurally open to accommodate agents’ diversity and value pluralism; thus, they are most profitably able to handle epistemic and moral disagreement. Mainstream approaches in decision theory (Arrow 1963) or in ethical–political theory (i.e., Rawls’ reflective equilibrium)^22^ cope with value pluralism and possible disagreements in different ways: respectively, by fictionally presupposing value-freedom or value homogeneity or by postulating a practical reason capable of solving value conflicts in every situation. Nevertheless, cases such as of the “three children and a flute” (Sen 2009, p. 32) and “Ashraf’s hard choice” (Sen 2018, pp. 12–3) clearly demonstrate that more than one ethical view (and related foundational values) can be defensible and that, in several occasions, moral disagreement can hardly be eluded. A main insight of Sen’s reflection is that value or valuation disagreement does not represent a problem for rational social choice and valuation in general (as formerly supposed) but rather it testifies to the irreducible plurality and incommensurability of diverse agents’ values, rational goals and preferences that can coexist in the same deliberative situation. This condition is rather common in real choices but, theoretically, its possibility has been denied by the standard criteria of rational social choice widely accepted by both mainstream economics and liberal thinking. Sen (and other social choice theorists) substantially contribute to the review of the standard rational choice theory and related limitations concerning values.^23^ The resulting pluralistic interpretation challenges usual standards of rationality but also traditional interpretations of objectivity-impartiality, objective value judgements and objective knowledge in social valuation, public reasoning, collective choice, as well as scientific knowledge production.^24^ The concept of *positional objectivity* or *parametric dependence* (Sen 1993) is illustrative of a pluralist social perspective in which ethical reasoning or scientific inquiry does not require abstraction from the peculiarities of the subjects expressing value judgements, while agents’ plural values and goals can systematically be considered and accommodated. It represents a main source of distinctiveness among Sen’s accounts and other virtue-ethics approaches,^25^ especially in light of determining a *hierarchy of objective values* and similarly, the suitable operational strategies to meaningfully elicit “aggregate” social/public value. Precisely, “objectively good” ethical values and normative ideals that can suitably be translated into design requirements, embodied by products/artefacts and adopted to justify technological innovation in democratic settings, ought to be the ones surviving a public critical discussion and reasoned/rational interpersonal scrutiny and deliberation. Analogous deliberative methods that similarly imply overcoming the neopositivist fact-value dichotomy and claim for the actual possibility of *intersubjective rational deliberation* on values have been successfully defended by Thomas Scanlon and Hilary Putnam (In Sen 2009, pp. 33–35). A VSD grounded on Sen’s capability approach procedural-deliberative tenets—embodying formerly described conceptions of rationality and objectivity—enables technological design to be the product of collective and cooperative ethical reasoning and principled reflection. The plurality of (non-epistemic) values of ethical and social importance that are embodied technologically are objective (interpersonally objective) thus, conceivably, easier to be converted into an essential source, rather than a constraint, for technological innovation and development in concrete social contexts. In other words, Sen’s procedural-deliberative account based on capabilities represents an original *pluralistic* and *realist* solution to the *moral overload* problem.

Third. The main empirical challenge entailed by a procedural-deliberative approach is to identify an objective-impartial procedure (based on the tenets above) suitable to elicit values underlying VSD in concrete technological design cases and social circumstances. As acknowledged, stakeholders’ inputs, values and goals can be gathered in various ways, including focus groups, interviews, surveys and other qualitative or quantitative methods (Spiekermann 2015, p. 170). Here, it is pivotal that values, goals and vested interests of the highest possible number of interested stakeholders should be evocatively represented, as well as that vital democratic desiderata be fulfilled. This decisively points to a more extensive adoption of *participatory-deliberative* methods to extrapolate values and “aggregate” social/public value.^26^ In the social sciences and well-being research, they openly contrast most popular eliciting methods such as surveys merely sum-aggregating individual preferences to get a collective-social standpoint. In poverty analysis, methods to elicit value such as participatory planning have been explicitly related to Sen’s capability approach’s deliberative tenets (Alkire In Kaufman 2005). These same considerations are supposed to be valid also when searching for suitable empirical strategies to validate human value-based technological design for well-being in democratic settings. When moving to VSD, it is not difficult to imagine a conceptual phase made of *deliberative workshops* (based on a *focus groups research design*^27^) with representatives of local civil communities, local economic operators, public authorities, and scientists/engineers/designers as a feasible option to evade not only the shortcomings of expert-led, predefined lists of universal values, but also the ones of standard economics eliciting methods merely sum-aggregating individuals’ preferences (population surveys, marketing research etc.).^28^ In deliberative workshops aimed at identifying a coherent set of foundational values and/or “aggregate” social/public value, people are called to *cooperate*, *intersubjectively reflect* and *trade off* to solve conflicts related to their own diverse opinions, beliefs, and vested interests. Potential value dilemmas and disagreements are solved by engaging in a *curious*, *creative* and *empowering* interpersonal discussion and deliberation that ultimately brings about a (self-chosen) hierarchy of “shared” values and goals*.* In this process, main epistemic and aggregation bias and even the so-called naturalistic fallacy (i.e., confusing what is preferred with what is ethical) are immediately circumvented, since the *interpersonal critical* discussion and rational deliberation facilitate the uprising of an objective-impartial *collective ethical viewpoint*. As evidenced by Søren Harnow Klausen (2018, pp. 14–15), a deliberative-procedural approach to value and well-being, through involving a kind of *joint commitment* to a *collective know-how*, goes beyond individuals’ prudential reasons and is able to generate valid and reliable *ethical group knowledge* (Harnow Klausen 2015). Thus, values and goals designated by means of open discussion and rational deliberation could legitimately be intended as an expression of agents’ context-sensitive objective-impartial value judgements that most evocatively embody genuine *social preferences* (not a mere sum-aggregation of individuals’ ones). This outcome is equally valid both in micro and macro deliberative situations—namely both at the societal level or in small interested groups (alternative targets of Sen’s and Harnow Klausen’s reflection). Thus, similarly to other participatory methods (i.e., participatory design, inclusive design etc.), the final aim is to enact agents’ *positive freedom* and overall, *human flourishing* by targeting human diversity in a creative but also in a more responsive manner, since—differently from prudential or instrumentally oriented decisions—a comprehensive ethical standpoint presupposes *positive duties* among participants.^29^Another important advantage of deliberative strategies is that experts, scientists, designers and engineers could participate in the group procedures selecting values and goals that must be embodied technologically in quality of *informed* stakeholders. Thus, problems deriving from the lack of technical information or lack of alignment among desirable ethical-social values and goals with accessible and realistic technical solutions for the specific cases under examination are immediately tested and solved. In essence, engineers/designers, instead of merely attempting to embody external ethical value-principles-ideals belonging to a somewhat pre-defined ethical theory, could immediately assess that values and goals are compatible with realistic technological possibilities. In other words, main epistemic biases are minimised while a *collectively* and *objectively* chosen hierarchy of values and goals is immediately corroborated not only from an ethical-democratic standpoint, but also from a scientific-technical point of view. The resulting methodology is able to go beyond the ethical aspects, that could be constraining to technological progress, and reconciles vital ethical and scientific considerations. Similarly, ethical and scientific paternalism is structurally evaded by increasing the accountability of value-based technological design and innovation strategies before their actual construction or implementation. The resulting method is *ethically* and *socially relevant* but also *clear-cut* since it is adjusted to specific scientific settings, technological cases or research fields. It is the active participation of scientists/engineers since the conceptual phase what would increase substantially the possibility that the chosen values can actually be translated in *purposeful* ethical technologies and high-tech products and artefacts.

To conclude, along with listing capabilities, another operational problem (Sen 2004, pp. 78–79) is determining the *relative weight* and importance of the different items/capabilities in the assessments carried out—namely a valuation problem that could not be solved unless paternalistically or arbitrarily, as rival ethical theories have often accused. This directly relates to the epistemic and especially, the aggregation challenges denounced by Van de Poel (In Oosterlaken 2015, p. 236) as intrinsic to the methods applied to evaluate decision alternatives under scrutiny in economic evaluations of well-being, health, poverty and so on. Paradigmatic examples are either standard economics cost–benefit analysis or later multi-criteria and multi-factorial valuation typical in multidimensional welfare indicators research. By tradition, main operational-practical difficulties (comparison, aggregation) and their negative impact on results, have been resolved by supposing the identification of utility and well-being and the total commensurability of every aspect valuable for well-being in utility-monetary terms. That is, in order to get an “aggregated” result, qualitatively different aspects of “good” and “value” are annulled by applying one-dimensional, utility-based methods such as cost–benefit, cost-effectiveness, cost-utility analysis to any sort of decision problem. Analogously, in multidimensional-multicriteria assessments, attaching numerical values to multifaceted evaluative dimensions—the so-called weighting/value schemes—is the strategy applied to establish the relative importance, aggregate and compare qualitatively different aspects/dimensions of well-being/health (i.e., the valuable set of capabilities) thus, to get a unique result. Namely, an “aggregate” value of well-being for every population under exam that is further ranked to allow the prioritization of interventions (based on these data). In other words, the solution adopted to handle the complexity and plurality of the real social world is *quantification;* namely, an aggregation strategy yet assuming the total commensurability of good and value. Moreover, the practice of assigning weights is known to entail certain *arbitrariness* and to deliver *inconsistent* results. As obvious, all these problems are totally circumvented in qualitative deliberative workshops based on a focus groups research design since incommensurable values-capabilities, as well as “aggregated” social/public value, *naturally* emerge from small-sized and localised procedures based on face-to-face interpersonal critical discussion and deliberation. For the sake of the argument, it is also important to report that main aggregation problems underlying multidimensional assessments have been recently solved also at a macro level by adopting innovative quantitative methods. In the field of economic evaluation of well-being/health, a recently outlined *capability-based account* (Cenci and Hussain 2019) shows how the adoption of specific tools such as “robust” model evaluation and “robust” methods allows for meaningful comparisons of qualitatively different evaluative dimensions without the need for assigning numerical weightings. Namely, plural and incommensurable aspects of well-being/health do not need to be weighted and aggregated to be compared and to get reliable and cogent results. The same methodology has also been profitably applied to eliminate the *threshold setting* problem (Hussain et al. 2020), also mentioned by Van de Poel as main deterrent for the adoption of the capability approach (or any other multidimensional account or measure) in the technological design for well-being field. Thus, main empirical problems and operationalization challenges underlying multidimensional well-being assessments based on capabilities (both versions) seem no longer predominant, and the application of one version or another (Sen’s or Nussbaum’s) depends solely on normative decisions of designers/engineers or the specific needs of particular case studies. Even so, the study by (Cenci and Hussain 2019) also points out that many valuable theoretical and epistemic insights related to multidimensional assessments of well-being/health could be inevitably lost if adopting Nusbaum’s rigid, over specified list instead of Sen’s approach underlying procedural ethics ideal.^30^ Precisely, the complexity and plurality demanded by multifaced ethical assessments of technologies and plural value-based technological design for well-being correlate better with Sen’s version of the capability approach while valuable societal targets could better be achieved once its participatory-deliberative tenets are explicitly assumed and operationalized.

## Conclusions and Future Research

This paper attempted to offer a plausible answer to fundamental questions of the general philosophical debate regarding value-laden applied science and objective knowledge production in democratic settings that are highly relevant to VSD as well but that has been neglected by existing accounts.

Overall, the paper fundamentally argues that *ethical proceduralism*, instead of substantive ethical theories most commonly related to VSD, offers significant theoretical, epistemic and also, practical-operational insights once a *procedural* VSD would be applied to specific case studies. Its main advantage relies on satisfying simultaneously both scientific-technological standards (epistemic values) and ethical-democratic desiderata (non-epistemic values). It should be imperative in an applied science domain as technological design and in VSD as leading value-laden approach in the field. Epistemic and non-epistemic aspects should be *complementary* in scientific inquiry (i.e., value-oriented technological design) and in producing valuable technical knowledge (i.e., value-based technologies and high-tech products/artefacts). This is attainable once espousing a procedural ethics stance and an ethical framework consistent with it since, while a moral foundation is actually performed, likewise, the epistemic advantages of the “morally neutral” VSD such as its flexibility, versatility, objectivity-impartiality, wide applicability or generalization potential are profitably preserved.

The benefits of this interpretation have been insightfully illustrated by the case presented in (section "The Case: Insights from the VSD of a Humanitarian Cargo Drone"). It makes clear that merely following or prioritizing the values and goals of designers or most powerful stakeholders as well as applying predefined ethical frameworks based on universal values cannot profitably escape the challenges encountered by the standard “ethically unsubstantiated” VSD in practical applications. Moreoever, it elucidates that vital ethical ideals such as positive freedom, self-determination and/or democratic desiderata like transparency, legitimacy and accountability of both the ethical procedure and chosen values and goals remain unsatisfied. They are, in fact, largely incompatible with the espousal of substantive ethical theories, for instance, popular expert-led, objective-list theories pervading the ethical–political liberal thinking. Thus, the case offers a solid empirical starting point (not typically provided in theoretical appraisals) to defend a compelling argument in favor of a precise theoretical alternative. Namely, by evading any possible relativist objection*,* it decisively points—not arbitrarily—to ethical proceduralism and Amartya Sen’s procedural-deliberative version of the capability approach as the most suitable ethical view to validate VSD in contemporary complex democracies.

As acknowledged, Sen’s approach procedural-deliberative tenets represent an *exception* within the liberal-egalitarian tradition opposing utilitarian theories in the field of well-being theory/research, including with regard to Martha Nussbaum’s theory of capabilities. It offers a broader understanding of human well-being and social justice (as *human flourishing*) while its adoption would be able to circumvent recurrent problems in VSD (moral overload, naturalistic fallacy). Moreover, flaws frequently depicted as intrinsic to an undifferentiated “Capabilitarian” perspective (epistemic and integration challenges) that, up to now, impeded its definite adoption in the field of *technological design for well-being* are amended. As a matter of fact, once Sen’s procedural-deliberative framework is applied to validate VSD, most of the ethical dilemmas related to human diversity, value pluralism, value conflicts, epistemic-moral disagreements that are crucial in democratic settings, could better be handled. Likewise, Sen’s approach not only most fruitfully deals with the structural problems of liberal theories—ethical-scientific paternalism, moral relativism, objectivism about well-being—but also *unambiguously* indicates how an ethical framework in similar circumstances should be operationalized. That is, the values of ethical and social importance underlying VSD for well-being and/or the normative ideals behind value-laden applied sciences in specific social environments must be designated by means of *participatory-deliberative* methods. This strategy, further based on a “participatory science” ideal, resolves important ethical-democratic shortages since entails extending the participation to the design to a large variety of different stakeholders. Similarly, the scientific import of the collective choice procedure; namely, the *rigor* and *objectivity-impartiality* of the “ethical procedure” extrapolating values and principles validating VSD is preserved while the naturalistic fallacy problem is substantially minimalized. It has to do with the *kind of knowledge* that can be extrapolated by deliberative workshops, focus groups or any other experimental technique based on public debate and interpersonal critical scrutiny and deliberation. In keeping with later approaches in social epistemology and social choice theory, it is believed that although agent-value-context sensitive, the *ethical group knowledge* inferred is cogent, reliable and satisfactorily objective. This is precisely Sen’s capability approach’s procedural-deliberative tenets (and related methods) major contribution to VSD: an *objective-impartial ethical procedure* to select meaningful “shared” plural and incommensurable collective values and/or “aggregated” *social/public value* to validating technological design for well-being in concrete social settings. It is because of these ethical and epistemic insights that a VSD entailing a “deliberative” *conceptual phase* can convey on a *hierarchy of values* which can better contribute to superior both scientific and societal goals. All in all, Sen’s capability approach could contribute to a superior VSD for several reasons:

First, it stimulates public discussion and deliberation involving a larger variety of different stakeholders thus, the concern for all their plural-incommensurable values, rational goals of action and vested interests is fostered. Moreover, both economic efficiency and fairness-equity questions, asymmetrically important in mainstream ethical–political theories, are jointly addressed within the “ethics and economics” paradigm that is behind the comparative deontological-consequentialist framework based on capabilities. Here, social/public values validating science and technology are established on a case-by-case basis (i.e., agent-context dependent) and further tested in the light of the overall social outcomes arising from their actual implementation.

Second, in practical applications, a refined VSD espousing Sen’s approach evades the rigidity and over-specification of predefined, complete ethical-conceptual frameworks most usually applied to substantiate technological design for well-being practices. Indeed, even if committed to a specific ethical view (solving main value dilemmas), standard VSD’s intuitions regarding flexibility, versatility, adaptability to different contexts or case studies as well as its ability to produce, in an accurate and rigorous way, objective and reliable evidential knowledge is preserved and further enhanced. Sen’s procedural-deliberative tenets are what actually compensate the negative epistemic effects that a large tradition in applied sciences associate with the loss of value freedom/neutrality and that regularly led to deny the importance of (non-epistemic) ethical-social-political values (thus, the importance of VSD!).

Third, differently from standard ethical–political theories searching for universal values, Sen’s tenets hints for the adoption of participatory-deliberative methods in the design process that increase the concern for contextual specificities, perspectival values and social norms. But while not reducing VSD’s ability to be applicable in different settings or case studies; it augments the possibility of obtaining more adjusted analyses and policies.

Fourth, more practicable scientific and societal goals can be identified by assuring experts, scientists, designers (the ones applying VSD) a more fundamental role and participation since the VSD conceptual phase in which values and goals that need to be further embodied technologically are settled. Thus, the *alignment* between chosen values/goals and embodied technologies could be more “robust” and “evident”.

To conclude, by assuming attractive ideals of human value-based, participatory and citizens’ science, this paper has exposed how to plausibly “democratise” a technological design for well-being adjusted to concrete social environments and aimed at delivering highly *functional* likewise, *ethically relevant* and *socially justified* technologies and high-tech products/artefacts.

Intuitively, a procedural approach to ethics is much more demanding than an *ethical justification* based on pre-defined ethical frameworks grounded on a list of fixed universal values. Beyond the theoretical-methodological discussion provided by this paper, further theoretical and empirical work is needed to test and definitively establish the appeal and full potential of a refined VSD re-shaped in a procedural ethics terms and supplemented by Sen’s capability approach participative-deliberative tenets. A *capability-based procedural-deliberative* VSD inherently demands *multidisciplinary teams* working together on original case studies since the conceptual phases and throughout the entire design process applying the tripartite methodology in successive iterations. It rarely happens in practice and likely, it explains why a similar ethical view has been—up to now—scarcely considered as a preferential theoretical possibility for VSD or in ethics of technology more generally. In the future, perhaps also due to the insights provided by this paper analysis, is expected that more applied research in technological design for well-being would go in a similar direction.

## Footnotes

1 For example, Carrier (In Machamer and Wolters 2004, Ch. 14) argues that in applied science either avoiding bias regarding power and control or achieving essential scientific goals plainly demands scientists to concentrate on epistemic values and excluding non-epistemic ethical and social values. Evidently, a similar a view denies the importance of VSD.

2 An a*nti-positivism* attitude is often assumed in sociology of technology and/or in certain accounts of theory and methodology of the social sciences, for instance, Bruno Latour's extreme “social constructivism" and Bent Flyvbjerg’s “phronetic social science”. Comparing the main differences between our proposal for value-based technology and design and these interpretations exceeds the objectives of this paper.

3 Briefly, where the *positivist* believed that the goal of science was to uncover the truth, the *post-positivist critical realist* believes that the goal of science is to hold the goal of getting it right about reality, even though we can never achieve that goal.

4 Here, the term “paternalistically” is used to refer to choices that, either benevolently (ethical paternalism) or to guarantee scientific simplicity and efficiency (scientific paternalism), are taken by stakeholders/agents who are supposed to have the expertise or the authority to do so, often under the supposition of the general benefit. Discussing varieties of paternalism and their implications exceeds the objectives of this paper. For a detailed account of technological paternalism in the public arena, its variations and implications, see (Wong 2013).

5 The general validity of universal values is normally attached to intrinsic characteristics of the human beings.

6 This idea is taken by Van de Poel (2014) and the description he offers of the tool used to construct a set of root values that can aid bridging the cooperative design gap. In Van de Poel’s work, a top-down hierarchy of values consists of three distinct ranks, in which the higher-order ranks (objective values and norms) strongly influence the lower-order rank (design requirements). A main difference with the original account is the nature and scope of *objective value judgements* and strategies used to select the *objective values* at the top rank. In this paper, the emphasis is on the insights of ethical proceduralism and participatory-deliberative methods to deliver objective ethical knowledge satisfactorily; namely, a set of values suitable to substantiate VSD in contemporary complex democracies (full details are given in section "Technology and Design for well-Being: Constructing “Value” by Adopting Amartya Sen’s Capability Approach").

7 An overview of the tripartite, iterative VSD methodology, as intended by its creators to consist of a conceptual, an empirical and a technological phase, In (Friedman et al. 2013; Van De Poel 2014).

8 A *technically focused* version of the cargo drone re-design*,* carried out by applying the traditional VSD approach, is found in the conference paper by (Cawthorne and Cenci 2019) published in the proceedings of the *International Conference on Unmanned Aircraft Systems* (ICUAS), held in Atlanta (USA) in June 2019. This preliminary *technical work* illustrates how the WeRobotics company’s values and goals align better with different technical solutions. Namely, if VSD is explicitly performed, the designated values can produce a technological embodiment that would better satisfy the company’s goals and mission. Then, this re-design of the drone prototype inspired the current paper analysis which, differently from the original paper, explicitly addresses the theoretical and methodological limitations of the standard VSD in practical applications that were identified in the initial contribution.

9 The *insights* from the case has been used to extrapolate standard VSD underlying logic and to identify the theoretical-practical advantages of adopting a precise ethical view to substantiate its “morally free” tenets (main objective of the analysis). Accordingly, the decision of not doing any further empirical work in the Peru´s village (e.g., collecting data by engaging local stakeholders, in-depth analysis of socio-economic aspects etc.) relies on important epistemic and practical motivations. First, the WeRobotics project was concluded, thus, there was no opportunity to re-design the cargo drone and operate it in the same social and environmental circumstances. Second, operating a drone is not like repeating an experiment in a lab: there is a strict regulation to follow that changes substantially in different contexts. Future projects, inspired to the WeRobotics’ humanitarian cargo drone, will be based in the EU thus, the data collected in the Peru’s Amazon would have been unusable to re-build and operate a new drone in such a different context. Finally, yet due to the extreme socio-economic-environmental peculiarities of the context, findings (i.e., a set of specific values) would hardly be *generalizable* or *applicable* to explore and explain other cases (in another context and under different conditions).

10 Some kind of stakeholder engagement took place the weekend prior to the cargo delivery drone field tests both in person with relevant government officials, doctors, community representatives as well as via radio and by a newspaper article (Meier et al. 2018, pp. 11–12). It aligns with what recommended by the “Humanitarian UAV Code of Conduct” (UAViators 2016) in which *transparency* in communicating objectives and strategies to local communities is considered imperative. But locals were not directly involved in any way in the technological design of the drone.

11 The ethical framework chosen should not be intended as an *arbitrary choice* since the findings from the case allow to refute mainstream ethical frameworks and decisively point to a clear and definite alternative: ethical proceduralism and Sen’s Capability approach as the most suitable stance for ethical-democratic value-based technological design in contemporary complex societies/democracies. It reflects one of the main objectives of this paper (pp. 3–4): the choice of an ethical framework for VSD should be done as much “scientifically” as possible. For example, as it is done in this paper, by relying on the insights from the case in question.

12 Cummings (2006, pp. 702–4) supports twelve main values of ethical and social importance: human welfare, ownership and property, privacy, freedom from bias, universal usability, trust, autonomy, informed consent, accountability, calmness, identity, and environmental sustainability.

13 Martha Nussbaum’s version of the capability approach proposing a list of ten capabilities valuable for human life (2000, Ch. 2) and John Rawls’s five primary goods (1971, pp. 58–9) are popular objective list theories.

14 Differently from Manders-Huits (2011), it is believed that a standard procedure for identifying stakeholders—specific for VSD—is not at all necessary. Identifying stakeholders is an *empirical task* that is *totally independent* by any conceivable ethical foundation. As known in the empirical social sciences, the strategies adopted to identify stakeholders depend on the case, the settings, the level of controversy of the issues at stake etc. This is the reason why likely, a unique procedure applicable everywhere and in every situation is not even desirable. For a general explanation on how to carry out a stakeholder’s analysis, see (Varvasovszky and Brugha 2000). Even though their focus is on health policy the procedure illustrated can be applicable to many fields since is based on the fundamentals of empirical analyses engaging with people in concrete social settings. Further, on how to perform a stakeholder’s analysis dealing with power when identifying direct and indirect stakeholder’s that have different goals, unequal importance or influence in the decision-making regarding projects objective or how to realize them, see (Ryding Olson and Lindegaard Attrup 2015, Ch.4).

15 Similar conclusions have been reached by Umbrello (2019a, b). The first paper implicitly argues that the basic assumptions behind VSD are not sufficient to account for moral pluralism. The second one, explores how a more philosophically sophisticated VSD could better foster collaboration and cooperation between several stakeholders and engineers in the AI field.

16 For an overview of the ongoing debate regarding mainstream theoretical approaches in well-being research, see Reiss (2013, Ch. 12).

17 A comparison of the normative and meta ethical foundation in Nussbaum’s and Sen’s version of the capability approach, is found In Cenci (2011, pp.141–7). It is argued that Nussbaum’s list does not represent a novelty in contemporary ethical–political theory. Indeed, even if considering the list as “revisable” (Nussbaum 2000, p.105), it still suffers the shortcomings usually attached to mainstream liberal thinking (e.g., Rawls’s list of primary goods) in the fields of social welfare and redistributive justice: ethical/evaluative paternalism, ethnocentrism (emphasis on western liberal values), inattention to contexts, insensitivity to agents’ diversity and their specific needs.

18 The evident problems of Nussbaum’s list of ten capabilities; namely, paternalism, perfectionism and scarce concern for democracy has been repeatedly stated by Claassen 2011, 2014.

19 Moral problems, also in science and technology, often take the form of a moral overload: there are too many things that could be relevant from a moral point of view (safety, privacy, freedom, autonomy, fairness and so on) that would be impossible to fulfil all at once.

20 For an overview of the debate on the normative foundations of standard welfare economics and applied methodology, see Hausman and McPhersons (2007).

21 A discussion on whether or not VSD should rely on a set of universal values, see Borning and Muller (2012) and Friedman et al. (2013) In (Jacobs and Huldtgren 2018).

22 Reflective equilibrium is the decision method most extensively supposed in ethical–political theory (also by Nussbaum’s capability approach).

23 Sen (1977) provides one of the most meaningful ethical revisions of the value-free, individualistic notion of rationality adopted in economic theory (the one of the rational choice theory). Likewise, standard economics’ formal criteria of rational social choice – namely, completeness and transitivity – have also been challenged and, instead, incompleteness has been alternatively adopted to validate the value pluralism and incommensurability of the multiple valuable aspects of well-being (Sen 2018). The adoption of incompleteness is directly against normative theories providing complete lists of objects of practical value for human life (as Rawls, Nussbaum or other moral philosophers do).

24 The pluralistic view defended by this paper is consistent to several approaches in social epistemology; for an overview see Longino (2016).

25 For an overview of the main differences between Sen’s capability approach and other virtue-ethics approaches, see Van Staveren (2007) and Burbidge (2016).

26 One of the fields in which the empirical determination of social value has been considered particularly relevant is in public health analysis and policy. An example of empirical procedure to extrapolate social value and society’s preferences for fairness by means population health survey, see (Nord et al. 1999). The interpretation of social/public values supported in this paper aims to be an alternative.

27 An overview of when, how and why doing focus groups, In (Barbour 2007).

28 There is wide agreement with Van de Poel’s claim (2012 In Oosterlaken 2015) that standard economics eliciting methods generate epistemic bias. However, later research in the field of economic evolution of well-being/health (we cited in this end of this paper) evades many of the former epistemic and operational challenges. Well-being and health are evaluated by applying methods following the same logic and operational strategies thus, similar epistemic flaws apply to both research fields.

29 In Scalonian terms, the main *ethical task* in deliberative situations involving the larger possible number of interested stakeholders is for identifying what people reasonably “owes to each other”. The similarities with Sen’s account have been often evidenced, also recently, by Scanlon himself (2019).

30 The recent contribution to the overall debate concerning the valuation of capability in WB/health research offered by (Cenci & Hussain 2019) solves “pragmatically” the epistemological and aggregation problems described by Van de Poel (2012) as intrinsic to CA-based accounts and measures. Similarly, the epistemic flaws of multidimensional accounts of well-being/health based on “tout court” espousals of Nussbaum’s list of functionings (i.e., end states, actual realizations) are evidenced: too rigid, over specified and improperly conducing to a unique index of valuable capabilities. Conversely, Sen’s procedural-deliberative tenets and concretely, the idea of a wider “capability set” understood as “valuable options that can be chosen and not chosen by free agents “, is operationalized by applying the “robust” methodology. That is, aggregation and comparison problems are avoided while thousands different WB/health indexes are obtained. Here, multifaceted aspects of well-being/health related to personal, social and environmental circumstances, can alternatively be combined (Sen’s idea of *conversion factors*) to deliver more reliable pictures of people’s well-being/health in concrete or changing circumstances. It is expected to better exemplify the main concept of “capability” (i.e., alternative combinations of functionings) that can be intended in terms of people’s positive freedom as actual opportunity to achieve valuable self-chosen welfare or social justice realizations; in Sen’s words (1999, pp. 32–3), what free “people can have reasons to value”.

## References

[CR1] Arrow JK (1963). Social choice and individual values.

[CR2] Barbour R (2007). Qualitative research kit: Doing focus groups.

[CR3] Burbidge D (2016). Space for virtue in the economics of Kenneth J. Arrow, Amartya Sen and Elinor Ostrom. Journal of Economic Methodology.

[CR4] Cawthorne, D. & Cenci, A. (2019). Value sensitive design of a humanitarian cargo drone. In *2019 International conference on unmanned aircraft systems (ICUAS)* (pp. 1117–1125), IEEE.

[CR5] Cenci, A. (2011). Economía, ética y libertad en el Enfoque de las Capacidades. *Revista Laguna*, 29, 123–147. (Trad. ‘Economics, ethics and freedom in the Capability Approach’).

[CR6] Cenci, A. (forthcoming). The “economic method” and its ethical component: Pluralism, objectivity and values in Amartya Sen’s capability approach. In W. J. Gonzalez (Ed.) *Methodological prospect in scientific research: From pragmatism to pluralism.* Series Synthese Library, Springer Press.

[CR7] Cenci A, Hussain MA (2019). Epistemic and non-epistemic values in economic evaluations of public health. Journal of Economic Methodology.

[CR8] Claassen R (2011). Making capability lists: Philosophy versus democracy. Political Studies.

[CR9] Claassen R (2014). Capability paternalism. Economics and Philosophy.

[CR10] Comin F, Comin F, Fennell S, Anand PB (2018). Sens capability approach, social choice theory and the use of rankings. New frontiers of the capability approach.

[CR11] Cummings ML (2006). Integrating ethics in design through the value-sensitive design approach. Science and Engineering Ethics.

[CR12] Diekmann S, Peterson M (2013). The role of non-epistemic values in engineering models. Science and Engineering Ethics.

[CR13] Diekmann S (2013). Moral mid-level principles in modelling. European Journal of Operational Research.

[CR14] Dignum M, Correlje A, Cuppen E, Pesh U, Taebi B (2016). Contested technologies and design for values: The case of shale gas. Science and Engineering Ethics.

[CR15] Douglas H (2009). Science, policy, and the value free ideal.

[CR16] Fiksel J (2009). Design for environment: A guide to sustainable product development—eco-efficient product development.

[CR17] Friedman B, Kahn PH, Borning A, Huldtgren A, Doorn N, Schuurbiers D, Van de Poel I, Gorman ME (2013). Value sensitive design and information systems. Early engagement and new technologies: Opening up the laboratory.

[CR18] Harnow Klausen S (2015). Group knowledge: A real-world approach. Synthese.

[CR19] Harnow Klausen S (2018). Ethics, knowledge, and a procedural approach to wellbeing. Inquiry.

[CR20] Hausman D, McPherson M, Hausman D (2007). The philosophical foundations of mainstream normative economics. The philosophy of economics: An ANthology.

[CR21] Hussain, M. A. Siersbæk, N. & Østerdal, L. P. (Forthcoming 2020 *Social Choice and Welfare*). Multidimensional welfare comparisons of EU member states before, during, and after the financial crisis: A DOMINANCE APPROACH.

[CR22] Jacobs N, Huldtgren A (2018). Why value sensitive design needs ethical commitments. Ethics and Information Technology.

[CR23] Kaufman A (2005). Capabilities Equality: Basic Issues and Problems.

[CR24] Kitcher P (2001). Science, truth and democracy.

[CR25] Kitcher P (2011). Science in a democratic society.

[CR26] Longino HE (1990). Science as social knowledge: Values and objectivity in scientific inquiry.

[CR27] Longino, H.E. (2016). The social dimensions of scientific knowledge. In E. N. Zalta (Ed.) *The Stanford encyclopedia of philosophy*.Retrieved: https://plato.stanford.edu/entries/scientific-knowledge-social (20 April 2020)

[CR28] Machamer P, Wolters G (2004). Science, values and objectivity.

[CR29] MacIntyre A (2013). After virtue.

[CR30] Manders-Huits N (2011). What values in design? The challenge of incorporating moral values into design. Science and Engineering Ethics.

[CR31] Meier, P., Kloptocz, A., Curry, A. & Mason B. (2018). Cargo drone field tests in the amazon. https://blog.werobotics.org/wp-content/uploads/2017/10/WeRobotics-Report-on-Drone-Cargo-Field-Tests-Peru-2017.pdf. Last access 20 April 2020.

[CR32] Nord E, Pinto JL, Richardson J, Menzel P, Ubel P (1999). Incorporating societal concerns for fairness in numerical valuations of health programmes. Health Economics.

[CR33] Nussbaum M (2000). Women and human development: The capabilities approach.

[CR34] Nussbaum M (2006). Frontiers of justice: Disability, nationality, species membership.

[CR35] Oosterlaken I, Van den Hoven J, Vermaas P, Van de Poel I (2015). Human capabilities in design for values. Handbook of ethics and values in technological design: Sources, theory, values and application domains.

[CR36] Pols A, Spahn A, Van den Hoven J, Vermaas P, Van de Poel I (2015). Design for the values of democracy and justice. Handbook of ethics and values in technological design: Sources, theory, values and application domains.

[CR37] Rawls J (1971). The theory of justice.

[CR38] Reiss, J. (2013). *Philosophy of economics: A contemporary introduction*. Routledge Contemporary Introduction to Philosophy Series.

[CR39] Rice CM (2013). Defending the *objective list theory of well-being*. Ratio.

[CR40] Ryding Olson J, Lindegaard Attrup M (2015). Power in projects, programs and portfolios.

[CR41] Scanlon. T. M. (2019). Forms of hypothetical justification*. Journal of Human Development and Capabilities A Multi-Disciplinary Journal for People-Centered Development*, 127–133. Doi:10.1080/19452829.2018.1536970

[CR42] Sen A (1977). Rational fools: A critique of the behavioural foundations of economic theory. Philosophy and Public Affairs.

[CR43] Sen A (1985). Well-being, agency and freedom: The dewey lectures 1984. The Journal of Philosophy.

[CR44] Sen A (1987). On ethics and economics.

[CR45] Sen A (1993). Positional objectivity. Philosophy and Public Affairs.

[CR46] Sen A (2000). Consequential evaluation and practical reasoning. The Journal of Philosophy.

[CR47] Sen A (2004). Capabilities, lists, and public reason: Continuing the conversation. Feminist Economics.

[CR48] Sen A (2009). The idea of justice.

[CR49] Sen A (2018). The Importance of Incompleteness. International Journal of Economic Theory.

[CR50] Spiekermann S (2015). Ethical IT innovation: A value-based system design approach.

[CR51] Steen M (2013). Virtues in participatory design: Cooperation, curiosity, creativity, empowerment and reflexivity. Science and Engineering Ethics.

[CR52] Steen M (2016). Organizing design-for-wellbeing projects: Using the capability approach. Design Issues.

[CR53] UAVIATORS (2016) https://humanitariandronecode.files.wordpress.com/2017/12/uaviators-code-and-guidelines.pdf

[CR54] Umbrello S (2019). Imaginative value sensitive design: Using moral imagination theory to inform responsible technology design. Science and Engineering Ethics.

[CR55] Umbrello S (2019). Beneficial artificial intelligence coordination by means of a value sensitive design approach. Big Data and Cognitive Computing.

[CR56] United Nations. Sustainable developments goals. https://www.un.org/sustainabledevelopment/sustainable-development-goals/. Last access 20 April 2020.

[CR57] Tiles M, Oberdiek H (2005). Living in a technological culture: Human tools and human values.

[CR58] Van de Poel, I. (2010). Value sensitive design: Four challenges. https://www.slideshare.net/philengtech/ibo-fpetvsd. Last access 20 April 2020.

[CR59] Van de Poel, I. (2012). Can we design for well-being? In P. Brey, A. Briggle & E. Spence (Eds.). *The good life in a technological age*. London: Routledge.

[CR60] Van de Poel I, Michelfelder DP, McCarthy N, Goldberg DE (2014). Translating values into design requirements. Philosophy and engineering: Reflections on practice, principles and process.

[CR61] Van de Poel, I. & Royakkers, L. (2011). *Ethics, technology, and engineering: An introduction.* Wiley-Blackwell Publishing: London.

[CR62] Van den Hoven J, Himma KE, Tavani HT (2008). Moral methodology and information technology. The handbook of information and computer ethics.

[CR63] Van den Hoven J, Lokhorst GJ, Van de Poel I (2012). Engineering and the problem of moral overload. Science and Engineering Ethics.

[CR64] Van Staveren I (2007). Beyond utilitarianism and deontology: ethics in economics. Review of Political Economy.

[CR65] Van Wynsberghe A, Robbins S (2014). Ethicist as designer: a pragmatic approach to ethics in the lab. Science and Engineering Ethics.

[CR66] Van Wynsberghe A, Nagenborg M, Di Nucci E, Santoni De Sio F (2016). Civilizing drones by design. Drones and responsibility: legal, philosophical and socio-technical perspectives on remotely controlled weapons.

[CR67] Varvasovszky Z, Brugha R (2000). How to do (or not to do) a stakeholder analysis. Health Policy and Planning.

[CR68] Vermaas P, Kroes P, Van de Poel I, Franssen M, Houkes W (2011). A philosophy of technology: from technical artefacts to sociotechnical systems. Synthesis Lectures on Engineers, Technology and Society.

[CR69] WeRobotics (2019). https://werobotics.org/programs/. Last access 20 April 2020.

[CR70] Winkler T, Spiekermann S (2018). Twenty years of value sensitive design: A review of methodological practices in VSD projects. Ethics and Information Technology.

[CR71] Winner L (1980). Do artifacts have politics?. Daedalus.

[CR72] Wong PH (2013). Technology, recommendation and design: On being a ‘paternalistic’ philosopher. Science and Engineering Ethics.

